# Draft Genome Sequences of Histamine-Producing *Photobacterium kishitanii* and *Photobacterium angustum*, Isolated from Albacore (*Thunnus alalunga*) and Yellowfin (*Thunnus albacares*) Tuna

**DOI:** 10.1128/genomeA.00400-15

**Published:** 2015-04-30

**Authors:** Kristin Bjornsdottir-Butler, Susan A. McCarthy, Paul V. Dunlap, Ruth E. Timme, Ronald A. Benner

**Affiliations:** a FDA, Division of Seafood Science and Technology, Gulf Coast Seafood Laboratory, Dauphin Island, Alabama, USA; b University of Michigan, Ann Arbor, Michigan, USA; c FDA, Division of Public Health Informatics and Analytics, College Park, Maryland, USA

## Abstract

Histamine-producing bacteria are responsible for scombrotoxin (histamine) fish poisoning, a leading cause of fish poisoning in the United States. We report here the draft genome sequences of four histamine-producing (HP) *Photobacterium kishitanii* strains and nine HP *Photobacterium angustum* strains isolated from tuna.

## GENOME ANNOUNCEMENT

Scombrotoxin (histamine) fish poisoning (SFP) remains the leading cause of fish poisoning in the United States, despite efforts for its control (1). The illness is caused by ingesting fish containing high levels of histamine and other biogenic amines. Symptoms, which include flushing, sweating, nausea, headache, and diarrhea (2), occur within minutes to several hours after the consumption of toxic fish and generally subside within a few hours. Histamine is produced by naturally occurring bacteria capable of converting histidine in the fish to histamine by the action of histidine decarboxylase. Scombroid fish (e.g., tuna and mackerel) and other nonscombroid species (e.g., mahi-mahi and bluefish) that have naturally high levels of histidine in their muscle tissue are therefore susceptible to histamine formation (3).

Gram-positive and Gram-negative histamine-producing bacteria (HPB) exist, but the main HPB found in fish are Gram negative (2, 4). Previously, mesophilic HPB, such as *Morganella morganii*, *Raoultella planticola*, *Enterobacter aerogenes*, and *Photobacterium damselae*, were thought to be the most prevalent HPB in fish (5–7). However, there have been reports of psychrotrophic HPB (e.g., *Photobacterium phosphoreum* and *Morganella psychrotolerans*) isolated from fish that are capable of growth at refrigeration temperatures (8, 9). Some of these bacteria are indigenous to the fish, making the control of SFP challenging.

The *Photobacterium kishitanii* and *Photobacterium angustum* strains sequenced in this study were psychrotrophic HPB isolated from the anal vents of albacore (*Thunnus alalunga*) and yellowfin tuna (*Thunnus albacares*) from the North Pacific Ocean west of Hawaii. The strains were sequenced to confirm their identification and to characterize the histidine decarboxylase gene cluster.

The genomes were sequenced using the Ion PGM sequencer and Ion OneTouch 2 system with 400-bp reads (Life Technologies, Frederick, MD). Briefly, for DNA purification, single colonies were incubated in 5 ml of Luria 70% seawater (LSW-70) (10) at room temperature with shaking at 200 rpm for 24 h. DNA was extracted with DNeasy blood and tissue kits, according to the manufacturer’s instructions (Qiagen, Valencia, CA). DNA concentrations were determined using a Qubit 2.0 fluorometer with the Qubit double-stranded DNA (dsDNA) high sensitivity (HS) assay kits, according to the manufacturer’s instructions (Life Technologies). DNA was enzymatically fragmented using Ion Xpress Plus fragment library kits (Life Technologies), and size was selected with E-Gel SizeSelect 2% agarose gels in an E-Gel iBase unit (Life Technologies). The template for the Ion Torrent PGM instrument was prepared with Ion PGM Template OT2 400 kits and sequenced with Ion PGM sequencing kits on an Ion 314 Chip version 2, according to the manufacturer’s instructions (Life Technologies). For each isolate, the genomic sequence single-pass reads were *de novo* assembled using the SPAdes software (11) and annotated using the NCBI Prokaryotic Genomes Annotation Pipeline (http://www.ncbi.nlm.nih.gov/genome/annotation_prok/) (12). Through the annotation process, 4,566 to 4,603 and 4,274 to 4,442 genes were identified for the *P. kishitanii* and *P. angustum* isolates, respectively. The presence of the histidine decarboxylase gene was confirmed in all *P. kishitanii* and *P. angustum* isolates.

### Nucleotide sequence accession numbers.

The draft genome sequences of the four *P. kishitanii* and nine *P. angustum* isolates are available in GenBank under accession numbers JZSS00000000 to JZTE00000000.

## References

[1] Pennotti R, Scallan E, Backer L, Thomas J, Angulo FJ 2013 Ciguatera and scombroid fish poisoning in the United States. Foodborne Pathog Dis 10:1059–1066. doi:10.1089/fpd.2013.1514. 24093307

[2] Hungerford JM 2010 Scombroid poisoning: a review. Toxicon 56:231–243. doi:10.1016/j.toxicon.2010.02.006. 20152850

[3] FAO/WHO 2012 Joint FAO/WHO expert meeting on the public health risks of histamine and other biogenic amines from fish and fishery products. Food and Agricultural Organization of the United Nations, World Health Organization, Rome Italy http://www.fao.org/fileadmin/user_upload/agns/pdf/Histamine/Histamine_AdHocfinal.pdf.

[4] Landete JM, De Las Rivas B, Marcobal A, Muñoz R 2008 Updated molecular knowledge about histamine biosynthesis by bacteria. Crit Rev Food Sci Nutr 48:697–714. doi:10.1080/10408390701639041. 18756395

[5] Bjornsdottir K, Bolton GE, McClellan-Green PD, Jaykus LA, Green DP 2009 Detection of Gram-negative histamine-producing bacteria in fish: a comparative study. J Food Prot 72:1987–1991. 19777904 10.4315/0362-028x-72.9.1987

[6] Bjornsdottir-Butler K, Jones JL, Benner R, Burkhardt W 2011 Development of a real-time PCR assay with an internal amplification control for detection of Gram-negative histamine-producing bacteria in fish. Food Microbiol 28:356–363. doi:10.1016/j.fm.2010.06.013. 21356438

[7] Kim SH, Barros-Velazquez J, Ben-Gigrey B, Eun JB, Jun SH, Wie CI, An HJ 2003 Identification of the main bacteria contributing to histamine formation in seafood to ensure product safety. Food Sci Biotechnol 12:451–460.

[8] Emborg J, Dalgaard P, Ahrens P 2006 *Morganella psychrotolerans* sp. nov., a histamine-producing bacterium isolated from various seafoods. Int J Syst Evol Microbiol 56:2473–2479. doi:10.1099/ijs.0.64357-0. 17012582

[9] Kanki M, Yoda T, Ishibashi M, Tsukamoto T 2004 *Photobacterium phosphoreum* caused a histamine fish poisoning incident. Int J Food Microbiol 92:79–87. doi:10.1016/j.ijfoodmicro.2003.08.019. 15033270

[10] Dunlap PV, Jiemjit A, Ast JC, Pearce MM, Marques RR, Lavilla-Pitogo CR 2004 Genomic polymorphism in symbiotic populations of *Photobacterium leiognathi*. Environ Microbiol 6:145–158. doi:10.1046/j.1462-2920.2003.00548.x. 14756879

[11] Bankevich A, Nurk S, Antipov D, Gurevich AA, Dvorkin M, Kulikov AS, Lesin VM, Nikolenko SI, Pham S, Prjibelski AD, Pyshkin AV, Sirotkin AV, Vyahhi N, Tesler G, Alekseyev MA, Pevzner PA 2012 SPAdes: a new genome assembly algorithm and its applications to single-cell sequencing. J Comput Biol 19:455–477. doi:10.1089/cmb.2012.0021. 22506599 PMC3342519

[12] Klimke W, Agarwala R, Badretdin A, Chetvernin S, Ciufo S, Fedorov B, Kiryutin B, O’Neill K, Resch W, Resenchuk S, Schafer S, Tolstoy I, Tatusova T 2009 The National Center for Biotechnology Information’s protein clusters database. Nucleic Acids Res 37:D216–D223. doi:10.1093/nar/gkn734. 18940865 PMC2686591

